# Proof of concept of a smartphone app to support delivery of an intervention to facilitate mothers’ mind-mindedness

**DOI:** 10.1371/journal.pone.0220948

**Published:** 2019-08-22

**Authors:** Fionnuala Larkin, Janine Oostenbroek, Yujin Lee, Emily Hayward, Elizabeth Meins

**Affiliations:** 1 Department of Psychology, University of York, York, England, United Kingdom; 2 Department of Psychology, University of Virginia, Charlottesville, Virginia, United States of America; Temple University, UNITED STATES

## Abstract

The present study reports on the first evaluation of a parenting intervention utilizing a smartphone app, BabyMind. The intervention aimed to facilitate mothers’ *mind-mindedness*—attunement to their infants’ internal states. Mothers in the intervention group (*n* = 90) used the BabyMind app from their infants’ births and were followed up at age 6 months (*n* = 66). Mothers in the control group (*n* = 151) were recruited when their infants were age 6 months and had never used the BabyMind app. Mind-mindedness when interacting with their infants was significantly higher in intervention group mothers than in control group mothers. The intervention was equally effective in facilitating mind-mindedness in young and older mothers. These findings are discussed in terms of the potential for interventions utilizing smartphone apps to improve parenting and children’s developmental outcome in vulnerable and hard-to-reach groups.

## Introduction

Smartphone apps are increasingly being used to collect psychological data and deliver psychological interventions [1]. Apps have distinct advantages over face-to-face procedures: they are low-cost, already integrated into users’ lives, and very easily accessible. Many apps are targeted at parents and parents-to-be, with 1059 and 497 pregnancy-related apps listed on the iTunes and Google Play platforms respectively [2]. Given the increasingly ubiquitous ownership of smartphones, apps have the potential to provide support for parents on a grand scale, and may be particularly useful in reaching younger parents, for whom uptake of ante- and post-natal services is poor (e.g., [3]). However, no parenting app has yet been evaluated in terms of its efficacy in improving parenting behavior. The aim of the study reported here was to develop a parenting intervention that utilized a smartphone app to improve the quality of infant–parent interaction and provide the first such evaluation of a parenting app.

We chose to focus on attempting to facilitate a specific aspect of parenting: mind-mindedness [4]. Mind-mindedness is measured in terms of the extent to which the caregiver comments on the infant’s internal states in an appropriate (e.g., saying the infant likes the car if she smiles when she sees it) versus non-attuned (e.g., saying the infant is bored with the teddy when he is still actively engaged in playing with it) manner. Mind-mindedness is characterized by high levels of appropriate comments and/or low levels of non-attuned comments, with around a quarter of parents making no non-attuned comments [5, 6]. Zero scores for non-attuned comments—indicating that the caregiver never misinterprets the infant’s internal state—can be considered to be optimal.

Research over the past two decades has documented how caregivers’ early mind-mindedness is associated with a number of positive child outcomes (see [7] for a review). Appropriate mind-related comments in the first year of life predict secure infant–caregiver attachment [5, 8, 9], whereas non-attuned comments predict insecure attachment [6, 10]. Early appropriate mind-related comments are also predictive of superior mentalizing abilities throughout the preschool years [11–15].

Mind-mindedness has been reported to be related to maternal age, with lower levels of mind-mindedness in young mothers compared with their older counterparts [16]. Although no strong associations have been reported between families’ socio-economic status (SES) and mind-mindedness [17–21], some predictive relations between early mind-mindedness and children’s subsequent development have been found to be moderated by SES. Specifically in children from low SES backgrounds, appropriate mind-related comments predict fewer behavioral difficulties in the preschool years [22] and better performance in national standardized reading tests at ages 7 and 11 [23].

Given that both low SES and young motherhood are associated with poor child outcomes [24–29], increasing mind-mindedness in these groups may ameliorate the effects of social and economic deprivation on children’s development. Devising a method for improving mind-mindedness—particularly in a way that is appealing to young parents—thus appears worthwhile.

Parenting interventions delivered face-to-face are commonly aimed at improving parents’ capacity to read and respond to children’s emotions and mental states accurately (see [30] for a recent review). Intervention delivery methods are varied and can include elements such as individual therapy with mothers, child development education, and modeling how to take a reflective stance and engage sensitively with children [30, 31]. There is already research showing that it is possible to facilitate parents’ mind-mindedness through intervention. For example, a video-feedback intervention designed to facilitate mind-mindedness proved effective in increasing mind-mindedness in mothers hospitalized with a range of severe mental illnesses [32]. The intervention prompted mothers to consider what their infant was thinking or feeling at three specific moments during a short infant–mother interaction filmed on admission to hospital. It resulted in a marginally significant increase in mothers’ appropriate mind-related comments, and a highly significant decrease in non-attuned mind-related comments between admission and discharge. At discharge, the intervention group did not differ significantly from a control group of psychologically-well mothers on either appropriate or non-attuned mind-related comments. Moreover, compared with mothers who had received standard hospital care, mothers who received the intervention were more likely to have infants who were securely attached at follow-up in the second year of life.

Colonnesi et al. [33] also reported on a video-feedback mind-mindedness intervention. The intervention involved adoptive parents and children and entailed eight sessions over a period of 3 months. During the intervention sessions, parents were guided in how to name children’s behaviors and mental states in objective, non-judgemental, sensitive ways, akin to appropriate mind-related comments. The intervention was reported to reduce attachment insecurity in adoptive parent–child relationships.

Although effective, the labor-intensive nature of these mind-mindedness interventions means that they could not be delivered at scale. To address this shortcoming, the study reported here developed an intervention consisting of a brief, face-to-face psychoeducational session, coupled with an app (BabyMind) to facilitate mind-mindedness. We wanted the app to encourage parents to think about the world from their infant’s perspective, and to enable communication between app users and the research team. More specifically, we wanted the app to deliver daily alerts (a) to provide the parent with evidence-based information about infant psychological development that was tailored to the age of each user’s infant, and (b) to prompt the parent to post a photograph or video clip to indicate what the infant was thinking or feeling. Alerts in both (a) and (b) used the name of the parent’s own infant to reinforce viewing the world from their infant’s perspective. The research team viewed the material posted in response to (b) and commented to facilitate parents’ mind-mindedness.

**Fig 1:**
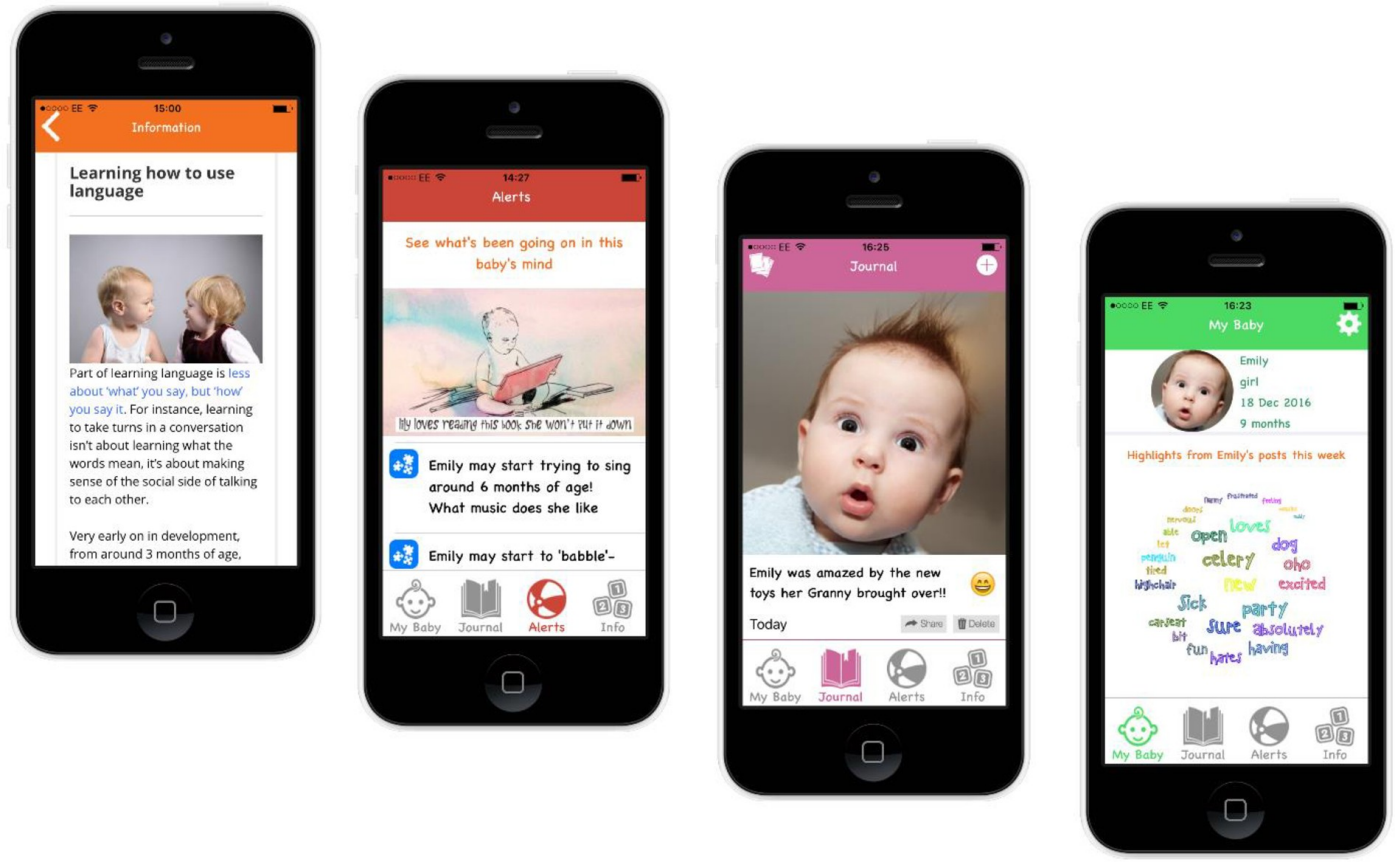
Screenshots from BabyMind showing Information Page, Alerts, Journal, and My Baby tabs.

For example, if a posted comment was deemed to be mind-minded (e.g., “Molly was surprised to see a squirrel” accompanied by a photograph of the infant looking surprised), the researcher replied in the first person from the infant’s point of view (e.g., “Oh, what’s that?”) or responded with a friendly comment, designed to provide reinforcement and positive feedback (e.g., “Great mind-reading!”). Where the comment was not mind-related (e.g., “We’re heading out to the shops”), the researcher responded with a further prompt for mind-mindedness (e.g., “How does Emily feel about shopping?”), or again modeled speaking on behalf of the baby (e.g., “Let’s go Mummy!”). The users viewed the research team’s response in the app, but could not comment further on the post. The two other tabs in the app consisted of a profile page and a link to a website developed for the current study with educational information on child development (see Fig 1). It was important for the app to be interesting and fun to use and to tap into commonplace aspects of modern parenting—sharing photographs and information about their children on a social media platform. Interventions that work within a parent’s comfort zone are less likely to be perceived as burdensome and are thus more likely to engage parents [34].

The main aim of the present study was to evaluate the efficacy of the intervention (face-to-face session plus BabyMind app), by comparing levels of appropriate and non-attuned mind-related comments in a group of mothers recruited in pregnancy who had used the app since birth (intervention group), and a group of mothers recruited when their infants were 6 months who had never used the app (control group). We hypothesized that intervention group mothers would show higher levels of appropriate comments and lower levels of non-attuned comments compared to control mothers. Given that previous research has reported that younger mothers produce fewer appropriate mind-related comments compared with their older counterparts [16], we investigated whether receiving the intervention would reduce or negate the previously observed relation between maternal age and mind-mindedness. We were therefore particularly interested in comparing young intervention group mothers with young and older mothers in the control group. We predicted that the young mothers in the intervention group would no longer show significantly different levels of mind-mindedness compared to older mothers. We also investigated relations between frequency of using the app and mothers’ mind-mindedness when interacting with their infants.

Finally, we assessed infant temperament in order to establish whether any group differences were independent of variability in infants’ temperamental characteristics. We did not make any predictions about infant temperament specifically, but included it to control for any differences in infant characteristics which may have influenced mother–infant interaction.

## Materials and methods

### Participants

The participants were recruited via local maternity services, community centers and events, parent–baby groups, social media, and word of mouth. The intervention group consisted of 90 women who completed their baseline assessment and began the intervention during the last trimester of pregnancy. Of these women, 66 returned for the follow-up assessment when infants (38 girls) were age 6 months. Although speaking to the infant in English was an inclusion criterion, one of the mothers spoke Polish to her infant, and her data were therefore excluded from the analyses. Of the women who returned for follow-up, 63 (95%) were White, 57 (86%) were in a relationship with the infant’s father, 8 were single, and 19 were aged 22 or younger. Attrition was due to mothers being too busy to continue (12), unable to be contacted (10), or losing custody of the infant (1), or the baby becoming too unwell to participate (1).

The control group of 151 mothers was recruited when women were either pregnant or had a young infant (72 girls); they did not receive the intervention, and were assessed for the first time when their infants were approximately 6 months of age. Of the 151 mothers, 145 (96%) were White, 134 (89%) were in a relationship with the infant’s father, 14 were single, and 30 were aged 22 or younger.

Women were only eligible to participate in the study if they owned or had regular access to a smartphone; three women did not meet this eligibility criterion. Testing was carried out in line with the guidance of the British Psychological Society and American Psychological Association. The study was approved by the National Health Service Health Research Authority Research Ethics Committee (REC reference: 14/NE/0114, IRAS project ID: 126036). All participants provided written informed consent to participate, and parental consent was also required for participating mothers who were age 15. All participants were paid £20 and travel costs per testing session, and the intervention group received an additional £10 gift voucher if they completed the follow-up session. In order to encourage young parents to engage with the BabyMind app, additional incentives were offered specifically to this group. Mothers were offered vouchers of increased value for posting 50 or more times (£15), 100 or more times (£20), or 150 or more times (£25).

When their infants were age 6 months, mothers in both groups provided demographic information on their ethnicity, age, relationship status, and highest educational level (from 1 = no qualifications and left school before age 16 to 7 = postgraduate degree), the infant’s date of birth, and the number of children they had (including the infant participating in the study).

### Intervention procedure

The women watched a 10-minute animated video to introduce them to the concept of mind-mindedness. After this, the experimenter modeled speaking on behalf of the participant’s unborn infant (e.g., “I’ve never heard that voice before…I wonder who Mummy is talking to?”). The women were asked to imagine what their unborn infants might feel and say when they are very active in the womb, and were encouraged to speak on the infant’s behalf. The experimenter emphasized the importance of ‘tuning in’ to the infant’s thoughts and feelings. Hypothetical situations were then described, and the women were asked to imagine what an infant might be thinking or feeling in these scenarios, and what they could say to the infant to show they understood his or her internal states. Given that this session took place during pregnancy, mothers-to-be did not receive any direct instruction on how to comment appropriately on their infants’ internal states because assigning mind-related comments as appropriate or non-attuned is only possible with reference to the infant’s actual behavior (see Mind-mindedness assessment below for details on coding mind-mindedness). Finally, the BabyMind app was installed on the participants’ smartphones and the functions were demonstrated to them. This demonstration focused on ensuring that participants knew how to use the app, and did not involve any direct teaching or guidance on the material. In total, the session (including the video) lasted between 15 and 20 minutes.

The app was designed by the research team and IC Mobile Lab in consultation with parents and maternity professionals. None of the authors have any financial stake in app. The ‘My Baby’ function allowed the user to personalize the app by adding their infant’s date of birth, name, and gender, and uploading a profile photograph. Intervention group mothers were asked to complete the ‘My Baby’ tab as soon as they could after the birth. The experimeter explained that the participant would receive a gentle text message reminder to use the app if they had not uploaded a post for one week, followed up by a call if there was still no activity within 3 days. Participants consented to this procedure.

The ‘Alerts’ function provided parents with evidence-based information about infants’ psychological development in the first year of life. A daily alert was programmed for each user to contain the individual infant’s name; these alerts provided facts about cognitive, linguistic, or socio-emotional development, and were all stored within the Alerts tab for future reference. The alerts were tailored to be relevant to the age of each user’s own infant. For example, “Maya will remember music she heard regularly when she was in the womb”, “Harry will understand what you’re saying well before he can speak–what words does he definitely know?” In addition, a weekly cartoon appeared as an alert, depicting an infant engaged in a common activity (e.g., looking at a book), with a caption that modeled what the infant might say if he or she could speak (e.g., “That was a good story–read it again!”). The aim of this function was to provide psychoeducation around infant development and mind-mindedness.

The app also contained an ‘Info’ function. This provided more content on infant psychological development across six domains: Forming relationships, Learning about the world, Learning to talk, Tuning into a baby’s mind, Ideas for play, and How do we study babies? Users were prompted to access these pages in their daily alerts, to find out more about specific topics. The purpose of these pages was to support users to hold developmentally-accurate expectations of their infants, and to provoke curiosity about the infant’s mental life.

The ‘Journal’ function used the same daily alert to prompt users to engage in mind-mindedness: “What’s on [Name’s] mind?” This was the cue for the user to upload a note, photo, or video to their Journal tab, documenting something their baby had been thinking or feeling that day. The tab’s text options included a wide range of emojis, and parents were informed that they could add these to their posts or simply post the emoji without any text. Note that parents were given no instructions about the type of material to upload in response to the daily prompt. The content of this page was uploaded to a secure server, where it could be accessed only by the study team. A member of the study team evaluated whether or not the user’s post was mind-related and responded accordingly. The study team’s response to the post was visible in the Journal tab, but users could not then comment on the team’s response; the cycle for each individual post ended with the researcher’s post. The user could start the cycle again with a new post on the Journal tab, but the app was specifically designed not to allow for extended back-and-forth interaction between the user and the research team. This meant that potential widescale individual differences in the amount of research team feedback provided to users were avoided. Words from the comments that each user uploaded were automatically extracted to generate a weekly word-cloud, which could be accessed on the My Baby tab. This was designed to encourage the user to reflect on the kind of week the infant had experienced (see Fig 1).

At the follow-up session when infants were age 6 months, mothers were asked to report on their use of the BabyMind app using a 4 point scale: 0 = rarely/never; 1 = a few times a month; 2 = a few times a week; 3 = every day. Note that these ratings included use of all functions of the app, not only posting material on the Journal tab.

### Mind-mindedness assessment

We measured maternal mind-mindedness from a filmed infant–mother interaction when infants were age 6 months. The interaction consisted of a 10-minute free-play session in the University’s developmental laboratory, which was equipped with a play-mat and a range of age-appropriate toys. The mother was instructed to play with her infant as she would if they had free time together at home. The interactions were later transcribed verbatim, and the transcripts were used in conjunction with the filmed observations to code the interactions for mind-mindedness [35].

Comments in which the mother referred to the infant’s internal state or spoke in the first person on the infant’s behalf (mind-related comments) were coded as either appropriate or non-attuned. A mind-related comment was coded as appropriate if (a) the researcher agreed with the mother’s reading of the infant’s current internal state (e.g., “That’s your favorite toy” in response to an infant repeatedly playing with the same toy), (b) the mother linked the infant’s current internal state to related events in the past or future (e.g., “Do you remember the duck you saw at the farm last week?” while the child was playing with a toy duck), (c) the comment was designed to engage the infant in play after a lull in the interaction (e.g., “Do you want to see this one?”), or (d) the mother voiced in the first person what the infant would say if s/he could talk.

A mind-related comment was coded as non-attuned if (a) the researcher judged the internal state to be inconsistent with the infant’s behavior (e.g., “You’re bored with that one” when the infant was actively engaged with playing with a toy), (b) the internal state was unconnected to the infant’s current activity (e.g., “You’d like to go to the playground after this”), (c) the mother tried to redirect the infant while he or she was already engaged in play (e.g., “Do you want the rings?” while the infant was playing with the bear and had shown no interest in the rings), (d) the mother seemed to project her own internal state on to the infant, or (e) the referent of the comment was unclear. Scores for appropriate and non-attuned mind-related comments were expressed as percentages of the total number of comments produced during the interaction to control for maternal verbosity.

The observation sessions were coded by a researcher who was blind to whether the mother had received the intervention and to all other data. A second blind researcher coded a randomly selected 25% of the sessions; inter-rater reliability for coding mind-related comments as appropriate or non-attuned was κ = .80.

### Infant temperament

Infant temperament was assessed at 6 months using the car seat task from the Infant Laboratory Temperament Assessment Battery (Lab-TAB; [36]). This task has been shown to have good predictive and concurrent validity in measuring temperament when used in isolation from the full LabTAB battery, and has strong ecological validity [37, 38]. For this procedure, a confining car seat was located on an upright chair in front of a camera in the developmental laboratory. The mother strapped her infant in to the car seat without speaking, and then stood to the side and slightly behind the car seat (where the infant could see her by turning his/her head) and refrained from looking at the infant. Thirty seconds were timed from when the mother closed the buckle of the car seat. The camera recorded a close-up, frontal view of the child’s face and body in the car seat.

The 30-second period was divided in to six 5-second epochs, and each epoch was coded for the presence and intensity of behaviors indicative of frustration and sadness. One rater blind to hypotheses and group rated all of the recordings, and a second blind rater coded a quarter of the recordings. Inter-rater reliability for the ratings were: facial anger: ICC = .74; facial sadness: ICC = .77; distress vocalization: ICC = .85, and physical struggle: ICC = .81. Scores across the epochs were averaged to give mean scores for facial anger, facial sadness, distress vocalization, and physical struggle. There was good internal reliability for a composite measure of the four scores, Cronbach’s αlpha = .71; composite scores were therefore used in the analyses below. Higher scores indicated greater negative affect, distress, and struggle, and index more difficult temperament.

## Results and discussion

### Descriptive statistics and preliminary analyses

Temperament data were available for 207 infants. Missing data were due to the assessment being terminated due to high infant distress (n = 1), or the requisite testing equipment not being available or the mother not following the protocol instructions (n = 9).

The intervention and control groups were found to differ with respect to three background variables: (a) intervention group infants were younger than control group infants, (b) the number of children in the family was higher in the intervention group than in the control group, and (c) intervention group mothers were marginally less well educated than control group mothers (see Table 1). These variables were therefore controlled in the analyses below. As shown in Table 1, the groups did not differ with respect to maternal age or infant temperament.

**Table 1:** Descriptive statistics for demographic variables for the intervention and control groups.

	BabyMind *M* (*SD*) Range	Control *M* (*SD*) Range	*t* statistic	Effect size (*d*)
**Infant age (weeks)**	25.85 (2.14) 20–32	28.55 (4.29) 21–48	4.86**	.84
**Maternal age**	28.52 (7.12) 15–44	29.60 (6.64) 16–47	1.08	.16
**Maternal education**	5.00 (1.52) 2–7	5.38 (1.59) 2–7	1.66^a^	.24
**Number of children**	1.41 (0.63) 1–3	1.24 (0.50) 1–3	2.13*	.30
**Infant temperament**	2.93 (2.23) 0.17–8.83	2.99 (2.24) 0–9.17	0.16	.03

^a^*p* < .10

*p < .05

**p < .001

Replicating previous findings [6, 39, 40], appropriate and non-attuned mind-related comments were unrelated in both the intervention, *r*(63) = .18, *p* = .145, and control, *r*(149) = .10, *p* = .247, groups. In the control group, neither appropriate nor non-attuned comments correlated with infant age, number of children in the family, or maternal education (*r*s < .15). In the intervention group, these correlations were similarly non-significant (*r*s < .16), apart from a negative correlation between non-attuned mind-related comments and maternal education, *r*(63) = -.27, *p* = .029. Infant temperament was unrelated to appropriate mind-related comments in both the intervention, *r*(61) = .13, *p* = .296, and control, *r*(141) = .12, *p* = .171, groups, and was unrelated to non-attuned mind-related comments in the control group, *r*(141) = .12, *p* = -.07, *p* = .392. These null findings replicate those of previous research that assessed temperament using maternal report [21]. However, non-attuned mind-related comments were positively correlated with infant temperament scores in the intervention group, *r*(41) = .28, *p* = .025, indicating an association between non-attuned mind-related comments and difficult temperament.

### Use of the BabyMind app

Self-reported app usage data were available for 64 mothers: one respondent left this item blank on the feedback form and the other mother did not complete this form due to experimenter error. Mothers’ modal response to how frequently they had used the app overall was “a few times a week”, with 30 mothers (48.4%) choosing this option; 11 (17.2%) users reported that they used the app every day, with only four (6.1%) users reporting that they rarely or never used the app.

The frequency of use of the BabyMind app was calculated specifically in relation to posts on the Journal tab; these data were available for all 66 participants. The number of posts across the 6-month period was *M* = 58.35 (*SD* = 41.34, Range = 1–165, median = 43.5). Older mothers (*M* = 63.26, *SD* = 41.14) did not differ from younger mothers (*M* = 46.21, *SD* = 40.33), in frequency of posting, *t*(64) = 1.53, *p* = .130. The number of journal posts was highly positively correlated with self-reported overall use of the app, *r*(64) = .69, *p* < .001.

### Young mothers’ levels of mind-mindedness

Participants were divided into groups on the basis of whether they were 22 years of age or younger, based on consistent findings linking childbirth under 23 with poorer outcomes for mothers and children [25, 29, 41]. In order to compare our results with those of Demers et al. [16], we investigated whether mean scores for appropriate mind-related comments differed between the younger and older control group mothers. (Note that this analysis was conducted only on the control group mothers to avoid any potentially confounding effects of receiving the intervention; the data below thus differ from those reported in Table 2).

**Table 2:** Mind-mindedness variables as a function of age and group.

	BabyMind		Control		Younger		Older	
	n = 66		n = 151		n = 49		n = 168	
	*M* (*SD*)	Range	*M* (*SD*)	Range	*M* (*SD*)	Range	*M* (*SD*)	Range
**Appropriate MRC (%)**	8.94 (5.50)	0–33.33	3.98 (2.57)	0–11.59	5.56 (5.85)	0–33.33	5.45 (3.80)	0–18.83
**Non-attuned MRC (%)**	0.77 (1.23)	0–7.32	2.45 (2.05)	0–8.93	2.14 (2.38)	0–8.93	1.89 (1.88)	0–8.42

*Note*: MRC = mind-related comments. % = number of appropriate or non-attuned MRC as a percentage of all comments made during the interaction.

Partially replicating the significant age-related difference reported in this previous study, there was a strong trend for younger mothers (*M* = 3.19, *SD* = 2.44) to have lower scores for appropriate mind-related comments than their older counterparts (*M* = 4.18, *SD* = 2.57), *t*(149) = 1.91, *p* = .058, *d* = .40. Demers et al. did not report on non-attuned mind-related comments, but the present study found no difference between the younger (*M* = 2.70, *SD* = 2.48) and older (*M* = 2.39, *SD* = 1.94) mothers’ scores for non-attuned comments, *t*(149) = 0.75, *p* = .456, *d* = .14.

### Differences in mind-mindedness between intervention and control group mothers

**Fig 2:**
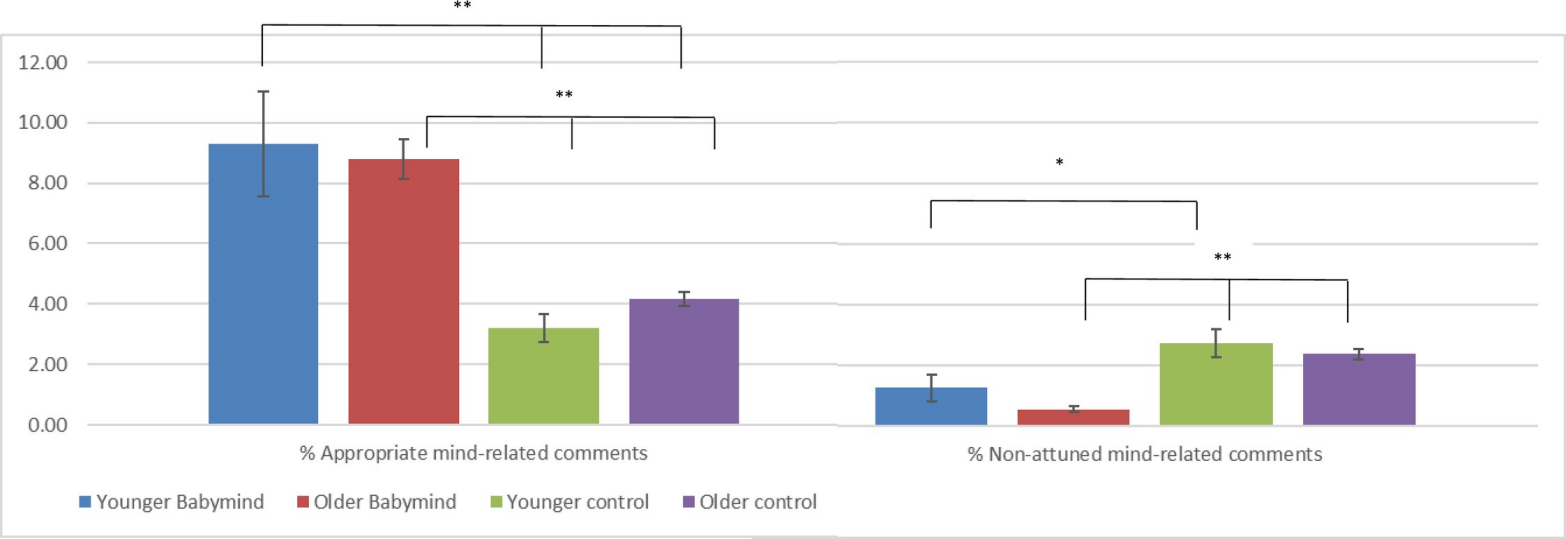
Means for proportions of appropriate and non-attuned mind-related comments for young and older BabyMind and control group participants. * *p* < .05, ** *p* < .001.

Table 2 and Fig 2 show the mind-mindedness scores for the different groups. The efficacy of the intervention in facilitating mind-mindedness was tested using MANCOVA, with group (BabyMind, control) and age (younger, older) added as fixed factors, infant age, number of children, and maternal education added as covariates, and scores for appropriate mind-related comments and non-attuned mind-related comments during infant–mother interaction at age 6 months added as the dependent variables. There was a main effect of group for both appropriate mind-related comments, *F*(1, 208) = 62.65, *p* < .001, η^2^ = .210, and non-attuned mind-related comments, *F*(1, 208) = 26.73, *p* < .001, η^2^ = .107. These differences represent large effects. There was no main effect of age for appropriate, *F*(1, 208) = 0.98, *p* = .324, η^2^ = .003, or non-attuned, *F*(1, 208) = 1.85, *p* = .176, η^2^ = .002, comments, and no group x age interaction for appropriate, *F*(1, 208) = 1.34, *p* = .244, η^2^ = .004, or non-attuned, *F*(1, 208) = 0.24, *p* = .625, η^2^ = .000, comments.

The results of the MANCOVA showed that younger and older mothers were equally likely to benefit from using the app. In order specifically to explore whether the previously observed relation between young maternal age and lower levels of mind-mindedness was eliminated in the intervention group, we compared the mind-mindedness scores of younger mothers who received the intervention with those of younger control mothers and older control mothers. Post-hoc pairwise comparisons following one-way ANCOVA showed that (a) for appropriate mind-related comments, the younger BabyMind group scored significantly higher than both the younger (*p* < .001) and older (*p* < .001) control group mothers, and (b) for non-attuned mind-related comments, the younger BabyMind group scored significantly lower than the younger control mothers (*p* = .035), with a non-significant trend (*p* = .069) for younger BabyMind group mothers to score lower than older control mothers (see Fig 2).

Since zero scores for non-attuned mind-related comments are considered to be optimal, we investigated whether age and receiving the intervention related to mothers never making a non-attuned comment. Zero scores for non-attuned mind-related comments across the four groups were as follows: seven mothers (36.8%) in the younger–BabyMind group, 24 mothers (52.2%) in the older–BabyMind group, six mothers (20.7%) in the younger–control group, and 12 mothers (9.8%) in the older–control group. Mothers in the intervention group were more likely to score zero for non-attuned comments compared with their control group counterparts, χhi^2^(1) = 32.27, *p* < .001, but there was no difference in zero scores for younger and older mothers, χhi^2^(1) = 0.57, *p* = .452.

### Frequency of app use and mind-mindedness at outcome

Self-reported app usage data were ordinal. The pattern of findings was identical when data were analyzed using parametric and non-parametric correlations; parametric correlation coefficients are reported for ease of interpretation of effect sizes. Self-reported frequency of app usage was negatively correlated with non-attuned mind-related comments *r*(62) = -.25, *p* = .048, but was unrelated to appropriate mind-related comments, *r*(62) = .11, *p* = .383. These data suggest that the more often users used the app overall, the fewer non-attuned comments they made while interacting with their infants. Given that only four mothers reported using the app rarely, it was not possible to perform group comparisons to investigate how reported app usage related to the mind-mindedness indices, so descriptive statistics as a function of self-reported usage are included here for information only (see Table 3).

**Table 3:** Mind-mindedness variables as a function of self-reported app usage.

	Rarely		A few times a month		A few times a week		Daily	
	n = 4		n = 18		n = 30		n = 11	
	*M* (*SD*)	Range	*M* (*SD*)	Range	*M* (*SD*)	Range	*M* (*SD*)	Range
**Appropriate MRC (%)**	7.68 (3.20)	3.76–11.38	8.09 (5.64)	0–18.83	9.15 (6.23)	0.45–33.33	9.63 (4.28)	2.92–18.18
**Non-attuned MRC (%)**	2.36 (1.74)	0.76–4.76	0.79 (1.69)	0–7.32	0.63 (0.84)	0–3.42	0.53 (0.81)	0–1.95

*Note*: MRC = mind-related comments. % = number of appropriate or non-attuned MRC as a percentage of all comments made during the interaction.

The frequency of posts on the Journal tab was unrelated to appropriate mind-related comments, *r*(65) = .10, *p* = .447, and non-attuned mind-related comments, *r*(65) = -.10, *p* = .428.

The results of the study reported here provide the first scientific evaluation of the efficacy of a parenting intervention utilizing a smartphone app, and suggest that the intervention was effective in facilitating mind-mindedness. When interacting with their 6-month-olds, mothers in the intervention group produced significantly more appropriate mind-related comments and significantly fewer non-attuned mind-related comments than control group mothers. These group differences held when demographic factors that were found to differ between the intervention and control groups were controlled. Mothers in the intervention group were more likely than their control group counterparts never to use a non-attuned mind-related comment. The groups did not differ with respect to infant temperament, suggesting that variability in the infants’ temperamental characteristics cannot explain the observed group differences in mind-mindedness.

The intervention was found to be equally effective in facilitating mind-mindedness in younger and older mothers. The high level of mind-mindedness in the younger intervention group mothers is particularly noteworthy given previous findings indicating that young mothers are less likely than older mothers to comment appropriately on their infants’ internal states [16], a finding that was partially replicated in the present study’s control group. These findings suggest that the intervention helps to reduce the disadvantage in mind-mindedness typically associated with being a younger mother.

We also demonstrated that the more often users reported engaging with the BabyMind app, the fewer non-attuned mind-related comments they made during actual infant–mother interaction. Only four mothers reported rarely using the app, so statistical group comparisons to investigate how self-reported usage related to mind-mindedness were not possible. However, descriptive statistics indicated increasing levels of appropriate mind-related comments and decreasing levels of non-attuned mind-related comments as a function of mothers’ self-reported use of the app. The same was not found for posting frequency on the Journal tab, with overall number of posts across the 6-month period being unrelated to both appropriate and non-attuned mind-related comments.

It is important to contextualize the levels of mind-mindedness observed in our intervention and control groups with those of comparable samples reported in the extant literature to establish whether the intervention was associated with greater mind-mindedness. The sample involved in Meins et al.’s (e.g., [6, 22]) longitudinal study provides a good comparison to the groups participating in the study reported here, being of similar size and social and economic diversity. In Meins et al.’s previous sample, mothers on average scored 5.34% for appropriate mind-related comments and 1.58% for non-attuned mind-related comments. These figures can be compared against 8.94% for appropriate and 0.77% for non-attuned mind-related comments in the intervention group, and 3.98% for appropriate and 2.45% for non-attuned mind-related comments in the control group. The difference between our intervention group and Meins et al.’s previous sample represents a large effect (*d* = .79) for appropriate comments and a medium effect (*d* = .52) for non-attuned comments. These comparisons clearly suggest that receiving the intervention was associated with higher scores for appropriate comments and lower scores for non-attuned comments.

Despite these differences between the intervention and control groups, intervention group mothers on average devoted less than 10% of their speech to commenting appropriately on their infants’ internal states, with the highest observed proportion of appropriate mind-related speech being a third. If the intervention was effective, should higher levels of appropriate mind-related comments have been seen? Research has not yet characterized the ideal proportion of speech that should be devoted to commenting in an appropriate manner on the infant’s thoughts and feelings, but we argue that the average level of these comments observed in the app users approximates to what one would hypothesize to be optimal. Caregiver speech in the first year of life obviously has a number of crucial functions—object labeling, describing events, asking questions, playing games, comforting and soothing, making the infant laugh—that would be neglected if caregivers spent the majority of the time commenting on the infant’s internal states. In contrast, zero scores for non-attuned mind-related comments can be regarded as optimal. The fact that such a high percentage of BabyMind users never made a non-attuned mind-related comment indicates that that our intervention was associated with mothers achieving the ideal for this index of mind-mindedness.

The BabyMind app did not directly instruct mothers how to comment appropriately on their infants’ internal states; rather, it informed them about psychological development relevant to the age of their infant and prompted them to consider what the infant was thinking or feeling at a specific time each day. Furthermore, while interacting with their infants in the developmental laboratory, mothers were not aware of which aspects of the interaction were of interest to the researchers. It is possible that the face-to-face session at baseline induced mothers to assume that we were interested in comments about their infants’ thoughts and feelings. However, it seems likely that self-consciously using mental state terms in the belief that this was the point of the study would result in high levels of both appropriate and non-attuned mind-related comments. In contrast, our findings showed that non-attuned comments were virtually eliminated in the intervention group. Intervention group mothers had not been instructed or coached on the difference between appropriate and non-attuned comments either during the initial session or via the app, so the fact that the intervention was associated with high scores for appropriate comments and low scores for non-attuned comments suggests that these mothers were genuinely ‘tuning in’ to their infants’ thoughts and feelings.

Previous interventions to facilitate mind-mindedness have used labor-intensive video-feedback methods, delivering the intervention on an individual basis [32, 33]. Our results suggest that mind-mindedness is amenable to change via an intervention that uses new technology with minimal face-to-face contact with parents. We designed BabyMind to capitalize on activities that were already part of everyday family life—parents taking photographs or video clips of their infants and posting them on an Internet platform. An important objective was to avoid making mothers feel that their parenting was being judged or corrected by the intervention. The app was thus designed to be interesting, accessible, and fun to use.

The results of the study reported here should be interpreted in light of a number of limitations. First, it is important to recognize that our aim was to establish initial proof of concept for the face-to-face session and BabyMind app as an effective parenting intervention. A crucial next step is to investigate whether the intervention can be demonstrated to be similarly effective in facilitating mind-mindedness in a more rigorous study, employing a randomized controlled design. Second, the present study cannot shed light on which precise aspects of the intervention—initial face-to-face session, psychoeducation, prompts to consider the infant’s mind, feedback from experts—were associated with facilitating mind-mindedness.

Interestingly, direct feedback from the research team does not appear to be the specific component of the BabyMind app that contributed to the effectivenes of the intervention. Feedback from the research team was given only in response to users’ posts on the Journal tab, and number of posts was found to be unrelated to mind-mindedness. The reason for this lack of association may be the fact that, by merely receiving the alert—regardless of whether it led them to post material—mothers could not help but reflect on their infant’s internal state. In contrast, self-reported usage of the app overall was associated with fewer non-attuned mind-related comments during mother–infant interaction.

A priority for future research is thus to explore which specific sub-components of BabyMind might facilitate mothers’ mind-mindedness. Due to technological limitations and privacy constraints we did not have access to objective measures of the frequency or duration of daily usage of the different tabs within the app. It would be useful to investigate the efficacy of the psychoeducation component or prompts to consider the infant’s thoughts and feelings in isolation, without any associated response from the research team. Exploring whether it is possible to train an artificial intelligence program to respond to parents’ posts in a manner that would facilitate mind-mindedness is also an interesting avenue for research. Alternatively, responses to posts in the Journal tab could be provided by other users rather than researchers. This is the model utilized in a large number of parenting apps, and many of the participants in the current study provided feedback indicating that they would enjoy this function. If future research could demonstrate that the BabyMind app is effective without its current function of providing expert feedback to parents, this would substantially increase its potential to deliver a parenting intervention on a grand scale.

It is also important to establish whether the initial face-to-face meeting with a researcher played a role in the success of the intervention. While our main purpose was to test the effectiveness of the BabyMind app, the intervention group received a brief face-to-face session at baseline to explain the concept of mind-mindedness. We therefore cannot rule out the possibility that the effect on mind-mindedness associated with receiving the intervention was an outcome of the face-to-face session. Meta-analytical data show that brief face-to-face interventions are effective in facilitating caregiver sensitivity and/or infant–caregiver secure attachment [42]. Interventions with fewer than 5 sessions were as effective as those with 5–16 sessions (both of which were more effective than those with >16 sessions). However, this meta-analysis also found that interventions involving very labor-intensive video feedback techniques were more effective than those using other methods. To establish the extent to which the very brief face-to-face session contributed to the efficacy of the intervention, levels of mind-mindedness could be compared in mothers-to-be who received only the face-to-face session or only the BabyMind app. To investigate this question, participants could be recruited online and download the BabyMind app themselves, without any researcher involvement at baseline. Future research could also investigate whether the intervention predicts more optimal infant–mother interaction over the longer term, as well as aspects of children’s future development.

## Conclusions

In summary, the present study demonstrated proof of concept of the intervention incorporating the BabyMind app, since mothers who had received the intervention and used the app from when their children were born were more mind-minded when interacting with their infants in the first year of life than control mothers. The intervention was equally effective in facilitating mind-mindedness in young and older mothers, and served to reduce the reported association between young motherhood and lower levels of mind-mindedness. It thus has the potential to be a highly cost-effective way of delivering the positive developmental outcomes associated with mind-mindedness to a large number of children, and may prove particularly useful for engaging hard-to-reach families or those who do not take up face-to-face services.

## Supporting information

S1 File Datafile. (SAV)
